# Binding of SARS-CoV-2 Nonstructural Protein 1 to 40S Ribosome Inhibits mRNA Translation

**DOI:** 10.1021/acs.jpcb.4c01391

**Published:** 2024-07-15

**Authors:** Hung Nguyen, Hoang Linh Nguyen, Mai Suan Li

**Affiliations:** † Institute of Physics, Polish Academy of Sciences, al. Lotnikow 32/46, 02-668 Warsaw, Poland; ‡ Quang Trung Software City, Life Science Lab, Institute for Computational Science and Technology, Tan Chanh Hiep Ward, District 12, Ho Chi Minh City 729110, Vietnam; § Institute of Fundamental and Applied Sciences, Duy Tan University, Ho Chi Minh City 700000, Vietnam; ∥ Faculty of Environmental and Natural Sciences, Duy Tan University, Da Nang City 550000, Vietnam

## Abstract

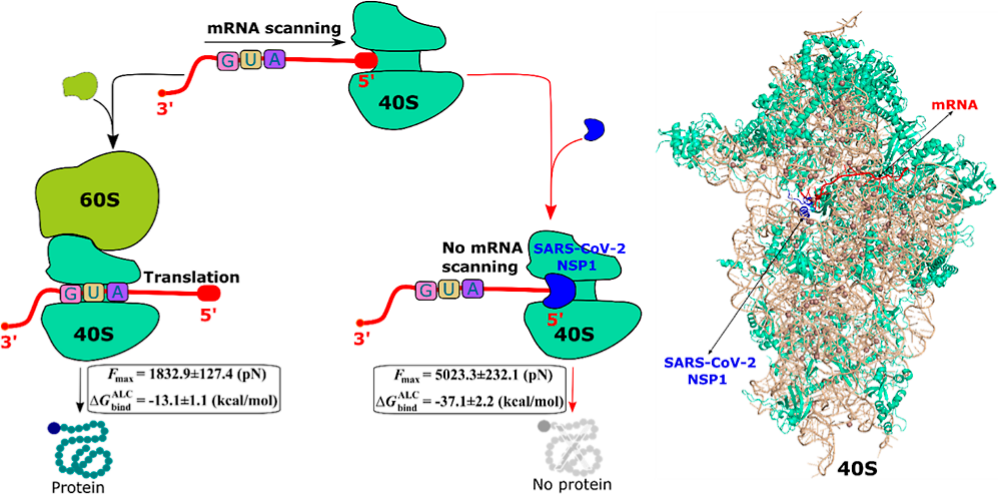

Experimental evidence has established that SARS-CoV-2 NSP1 acts as a factor that restricts cellular gene expression and impedes mRNA translation within the ribosome’s 40S subunit. However, the precise molecular mechanisms underlying this phenomenon have remained elusive. To elucidate this issue, we employed a combination of all-atom steered molecular dynamics and coarse-grained alchemical simulations to explore the binding affinity of mRNA to the 40S ribosome, both in the presence and absence of SARS-CoV-2 NSP1. Our investigations revealed that the binding of SARS-CoV-2 NSP1 to the 40S ribosome leads to a significant enhancement in the binding affinity of mRNA. This observation, which aligns with experimental findings, strongly suggests that SARS-CoV-2 NSP1 has the capability to inhibit mRNA translation. Furthermore, we identified electrostatic interactions between mRNA and the 40S ribosome as the primary driving force behind mRNA translation. Notably, water molecules were found to play a pivotal role in stabilizing the mRNA-40S ribosome complex, underscoring their significance in this process. We successfully pinpointed the specific SARS-CoV-2 NSP1 residues that play a critical role in triggering the translation arrest.

## 1 Introduction

Severe acute respiratory syndrome coronavirus 2 (SARS-CoV-2) caused the 2019 coronavirus disease (COVID-19) worldwide pandemic, which affected millions of people.^1^ Like other coronaviruses, SARS-CoV-2 is an enveloped, positive-sense, single-stranded RNA virus, and its closely related phylogenetic species are known to infect a large number of vertebrate species.^2^ The SARS-CoV-2 genome consists of about 30 kb linear, one of the 5′-capped and 3′-polyadenylated RNA genomic components that make up coronavirus particles, encoding two large overlapping open reading frames in gene 1 (ORF1a and ORF1b), and includes various structural and nonstructural proteins at the 3′ end.^3^ After entering host cells, the viral genomic RNA is translated by the cellular protein synthesis machinery to produce a set of nonstructural proteins that render cellular conditions favorable for viral infection and viral mRNA synthesis.^4^ In cells infected with SARS-CoV-2, one of the most enigmatic viral proteins is a host shutoff factor called nonstructural protein 1 (SARS-CoV-2 NSP1).^5^ SARS-CoV-2 NSP1 is the product of the N-terminus of the first open reading frame ORF1a and serves to suppress host gene expression and host immune response. Generally, SARS-CoV-2 NSP1 plays an important role in the viral life cycle.^6^

All viruses rely on cellular ribosomes for their protein synthesis and compete with endogenous mRNA for access to a translation machinery known as protein synthesis, which acts as a focal point of control.^7^ Host gene expression is limited by the common viral strategy of shifting translational resources toward viral mRNA.^8^ This phenotype termed host shutoff, increases the access of viral transcripts to ribosomes and promotes innate immune evasion.^8a^ Host shutoff is a hallmark of coronavirus infection and has significantly contributed to the suppression of innate immune responses in multiple pathogenic coronaviruses, including SARS-CoV, Middle East respiratory syndrome coronavirus, and pandemic SARS-CoV-2.^9^ SARS-CoV-2 induced host shutoff, which is multifaceted and involves inhibition of host mRNA splicing by SARS-CoV-2 NSP16, restriction of cellular cytoplasmic mRNA accumulation and translation by SARS-CoV-2 NSP1, and disruption of protein secretion by SARS-CoV-2 NSP8 and SARS-CoV-2 NSP9.^10^

**Figure 1:**
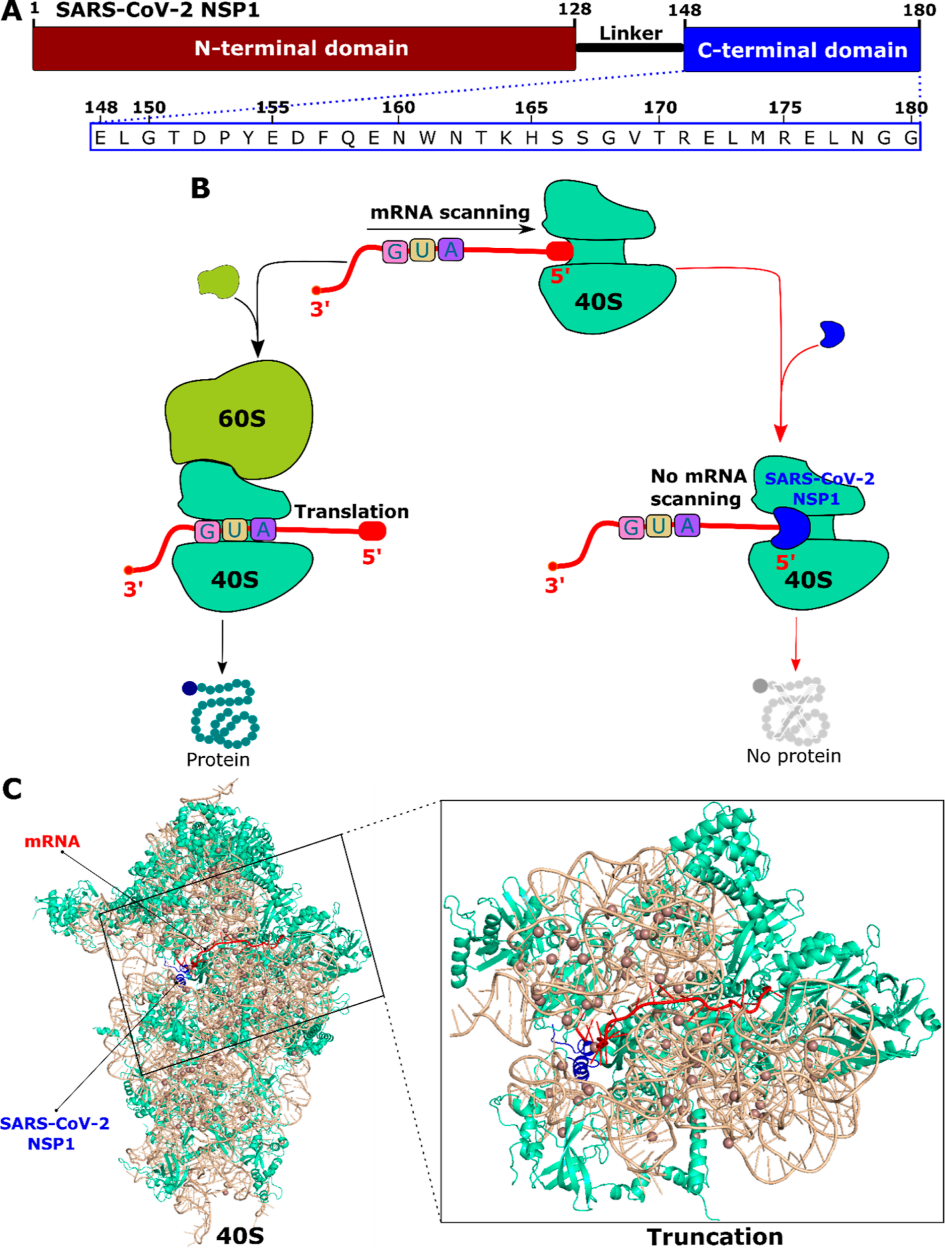
(A) Scheme of SARS-CoV-2 NSP1 structure; the C-terminal domain includes 32 residues from E148 to G180. (B) Scheme of SARS-CoV-2 NSP1 action to suppress mRNA translation (the mRNA sequence used in our simulations is C A G A C A C C A U G G U G C A C C U G A C). (C) 3D structure of the mRNA-40S-NSP1 complex constructed from a superposition of two different PDB structures 6ZOJ and 6HCJ and the truncation of the mRNA-40S-NSP1 complex. This structure includes rRNA (wheat), SARS-CoV-2 NSP1 (blue), mRNA (red), rproteins (green-cyan), and Mg^2+^ and Zn^2+^ ions (dark-salmon). The rectangle depicts a truncated ribosome used for the second set of simulation.

NSP1 of SARS-CoV (SARS-CoV NSP1) and SARS-CoV-2 NSP1 do not interact with 60S ribosomal subunit, they bind to only 40S ribosomal subunit and stall canonical mRNA translation at various stages during initiation.^11^ Although in vitro binding and translation assays revealed that both SARS-CoV NSP1 and SARS-CoV-2 NSP1 exert similar efficacy in the host translational shutdown mechanism,^12^ SARS-CoV-2 was shown to be more infectious and triggers more comorbid conditions than SARS-CoV.^13^ Here, SARS-CoV-2 NSP1 consists of 180 amino acids, which are organized into three distinct domains: the N-terminal domain, the linker domain, and the C-terminal domain (Figure 1A).^14^ An early model of SARS-CoV-2 NSP1 model lacks the C-terminal domain, as it remains disordered in solution and the ordered helix–loop–helix is formed only upon binding to the small ribosomal subunit.^12,14^ In contrast, the N-terminal and linker regions of SARS-CoV-2 NSP1 do not engage in direct binding to the 40S mRNA entry channel, but rather they are involved in stabilizing its association with the ribosome and mRNA.^12,15^ Schubert et al.^16^ recently showed that the C-terminal domain specifically interacts with the 40S subunit of the human ribosome, thereby causing inhibition of mRNA translation. It binds to the mRNA entry channel, folds into two helices, and interacts with h18 of the 18S rRNA (rRNA) as well as with the 40S ribosomal protein (rprotein) uS3 in the head and uS5 and eS30 in the body, where SARS-CoV-2 NSP1 would partially overlap with the fully accommodated mRNA. In short, SARS-CoV-2 NSP1 suppresses all cellular antiviral defense processes that depend on expression of host factors, including the interferon response. It acts as a ribosome gatekeeper to halt translation and inhibit host cell protein synthesis. This shutdown of key parts of the innate immune system may facilitate efficient viral replication^17^ and immune evasion. Its important role in dampening the antiviral immune response makes SARS-CoV-2 NSP1 a potential therapeutic target.^16,18^ However, the atomistic mechanism of how interactions between SARS-CoV-2 NSP1 and a conserved region in the 5′ untranslated region of viral mRNA suppress viral protein expression remains (Figure 1B).^16^

In computational work, Borišek et al.^19^ used all-atom simulation to investigate the interaction of SARS-CoV NSP1 and SARS-CoV-2 NSP1 to the 40S subunit of the ribosome. They found that upon SARS-CoV-2 NSP1/SARS-CoV NSP1 binding to 40S, the critical switch of Gln158/Glu158 and Glu159/Gln159 residues remodels the interaction pattern between SARS-CoV-2 NSP1/SARS-CoV NSP1 and neighboring proteins (uS3 and uS5) and rRNA (h18) lining the exit tunnel. This finding provides a clear picture of how SARS-CoV-2 invades human cells. However, the effect of SARS-CoV-2 NSP1 binding to 40S ribosome on mRNA translation has not been theoretically studied.

In this work, we applied steered molecular dynamics (SMD) and alchemical simulations to observe the effect of SARS-CoV-2 NSP1 binding to the 40S ribosome and inhibiting the mRNA translation process. Our results demonstrated that the presence of SARS-CoV-2 NSP1 significantly increased the binding affinity of mRNA to 40S ribosome, which means that SARS-CoV-2 NSP1 binding to the mRNA entry channel inhibits its translation in the ribosome. In addition, electrostatic mRNA-ribosome interactions have been found to play a key role in mRNA translation.

## 2 Materials and Methods

### 2.1 Building Two Complexes

To study the effect of SARS-CoV-2 NSP1 on the binding affinity of mRNA to the 40S ribosome, two complexes will be considered. One of them includes mRNA bound to the 40S ribosome and some additional components in the absence of SARS-CoV-2 NSP1, and this complex will be called mRNA-40S. The second complex, which will be referred to as mRNA-40S-NSP1, is similar to mRNA-40S, but in the presence of SARS-CoV-2 NSP1.

In detail, the cryo-EM structure of SARS-CoV-2 NSP1 in complex with the 40S ribosome and additional components including 18S rRNA, 60S rprotein L41, receptor of activated protein C kinase 1, and 165 Mg^2+^ and 2 Zn^2+^ions, was retrieved from the Protein Data Bank (PDB) with PDB identifier 6ZOJ.^16^ This structure is called 40S-NSP1 and was used as the basic for building the mRNA-40S and mRNA-40S-NSP1 complexes. The 3D structure of mRNA-40S-NSP1 was constructed by superimposing two PDB structures, 6ZOJ and 6HCJ,^16,20^ which means that the mRNA structure extracted from 6HCJ 20 was inserted into the 6ZOJ structure. The mRNA-40S was then obtained from the mRNA-40S-NSP1 by removing SARS-CoV-2 NSP1. The mRNA-40S-NSP1 complex is displayed by using the PYMOL package^21^ (Figure 1C). The divalent cations Mg^2+^ and Zn^2+^ stabilize rRNA, mRNA, and hence the ribosome complexes.

### 2.2 MD Simulations

Because the mRNA has been mechanically inserted into the complexes, they should be allowed to relax before running the SMD simulation. Since the systems are large they may not be equilibrated using only all-atom simulations forcing us to combine coarse-grained (CG) and all-atom simulations (see Supporting Information). First, we performed energy minimization, followed by a short 5 ns simulation in *NVT* and *NPT* ensembles, and a 1000 ns of conventional CG molecular dynamics (CGMD) simulation for mRNA-40S and mRNA-40S-NSP1 complexes using the MARTINI force field^22^ and CG water model.^23^ It should be noted that, due to the elastic network model implemented in the MARTINI force field, secondary structures are preserved during the simulation. However, using this MARTINI force field in the first step is acceptable because after mRNA insertion or NSP1 removal, the space around the entrance channel is needed to relax to accommodate molecules in this area, but does not care much about secondary structures. These structures are subject to change during the following all-atom conventional molecular dynamics (CMD) simulations.

The last snapshot of the CGMD simulation was converted to the all-atom structure and its energy was minimized by using the steepest-descent algorithm, followed by a short simulation of 3 ns in *NVT* and *NPT* ensembles, and then was a 500 ns production CMDs simulation for full 40S ribosome. By utilizing the clustering analysis on the snapshots obtained from a 500 ns all-atom CMD run, we were able to acquire10 representative structures. These structures were served as the starting point for conducting 10 independent SMD simulations. The most prevalent structure derived from clustering the snapshots obtained from the 500 ns CMD simulations of the mRNA-40S and mRNA-40S-NSP1 complexes was selected to carry out the MARTINI CG alchemical simulations. All steps of energy minimization and MD runs are described in Figure S1.

Additionally, to ensure that the full ribosome complexes are equilibrated we performed simulations for truncated mRNA-40S and mRNA-40S-NSP1. The structure of the most abundant snapshot obtained from the 500 ns CMD simulations of complete ribosomes was used for truncation. Truncated mRNA-40S and mRNA-40S-NSP1 are rectangular boxes with dimensions of 22 nm ≤ *x* ≤ 30 nm, 18 nm ≤ *y* ≤ 30 nm, and 7 nm ≤ *z* ≤ 19 nm (Figure 1C). The energy of these truncated complexes was then minimized, followed by short 5 ns simulations in both the *NVT* and *NPT* ensembles. A production all-atom CMD simulation of 1000 ns was carried out and the computational procedure was repeated as in the case of full ribosomes. Namely, from this run, 10 representative snapshots obtained by the clustering analysis were selected and used as initial structures for conducting 10 independent SMD simulations, while the most representative structure was used for MARTINI CG alchemical simulations. The purpose of this step was to compare the results obtained from the full 40S ribosome and the truncated 40S ribosome for mRNA-40S and mRNA-40S-NSP1. More details are shown in Figure S1. The AMBER99SB force field^24^ and the water model TIP3P^25^ were used for all-atom CMD runs for both full and truncated systems.

As shown in Figure S2, the root-mean-square deviation (rmsd) of both complexes exhibits fluctuations. The rmsd consistently remains below 0.35 nm in the CGMD simulations (Figure S2A). In the case of CMD simulations of the full ribosome, the rmsd reaches equilibrium after approximately 200 ns, displaying fluctuations around 1.15 nm (Figure S2B). Moreover, Figure S2B shows that NSP1 has little effect on the rest of the ribosomal complex structure. This is reasonable because our model only considers the C-terminal domain of NSP1, and this small fragment (32 residues), especially compared to the ribosome, may significantly affect the region near the mRNA entry tunnel, but not other parts of the ribosome. For CMD simulations of truncated complexes, equilibrium is also attained after 200 ns, with the rmsd fluctuating below 0.3 nm (Figure S2C). Thus, our results suggest that equilibrium was achieved in both the full and truncated models. Another reason to believe that these systems have already reached equilibrium in our simulations is that the PDB structure of the ribosome with NSP1 was used (PDB ID 6ZOJ). Addition of a short mRNA (and removal of the relatively short C-terminus of NSP1 to create the mRNA-40S complex) should not affect much the system. Of course, it is impossible to equilibrate ribosomal complexes starting from random conformations.

### 2.3 SMD Simulations

In order to probe the binding affinity of mRNA to the ribosome in the presence and absence of NSP1 SMD simulations^26^ were conducted by pulling it along its entry channel for full 40S and truncated 40S complexes. An external force is applied to the dummy atom connected to the 5′-mRNA (O5′ atom) through a spring with a stiffness *k*. In general, the direction of pulling is along the mRNA entry channel. The spring constant *k* was set to 600 kJ/(mol.nm^2^) (≈1020 pN/nm), which is a typical value used in atomic force microscopy experiments.^27^ The complexes were rotated so that the exit direction was parallel to the *z*-axis (Figure S3). A pulling speed of *v* = 0.5 nm/ns was used, and this value is about 10 orders of magnitude greater than in the experiment, but, as shown in previous works,^28^ this choice does not affect the relative binding affinity, i.e., it can be used to discern strong from weak binders. More details on SMD simulations can be found in Supporting Information.

### 2.4 Alchemical Molecular Dynamics Simulations

Since SMD at high pulling speeds only allows estimation of relative binding affinity, in order to evaluate the effect of SARS-CoV-2 NSP1 on the absolute binding affinity of mRNA to the 40S ribosome, alchemical free energy calculations were performed using the MARTINI CG model.

The mRNA-40S and mRNA-40S-NSP1 complexes used for alchemical free energy calculations were taken from the most populated structure for each system of 500 ns CMD simulations for the full 40S ribosome, and of 1000 ns CMD simulations for the truncated 40S ribosome. Here, the standard CG MARTINI 2.2 force field, which was developed for modeling of biological systems such as biological membranes, proteins, nucleotides, etc.^22,29^ was used to calculate the binding free energy of mRNA to the 40S and the 40S-NSP1. This force field is accurate enough to describe the ligand–protein, protein–protein, protein–DNA/RNA, and protein-liquid interaction in an aqueous medium.^22,29,30^ The MARTINI water model^23^ was used with a minimum distance between water beads of 1.0 nm. The system was neutralized by adding sodium chloride salt solution. The temperature was set to *T* = 300 K using a *v*-rescale thermostat,^31^ and pressure was set to *p* = 1.0 bar with a Parrinello–Rahman barostat.^32^ The LINCS algorithm^33^ was used to constrain the length of all bonds.

To evaluate the free energy of mRNA binding to the 40S ribosome with and without SARS-CoV-2 NSP1, we created the thermodynamic cycle described in Figure S4. From the thermodynamic cycle, we have
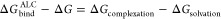
1Δ*G* ≡ 0 as it is related to noninteracting (λ = 1) mRNA being dummy and dummy-40S-NSP1. Then the binding free energy has the following form
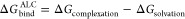
2

For alchemical transformations, we used an optimal set of λ-values ranging from λ = 0 to λ = 1, where λ = 0 and λ = 1 correspond to a system with and without full interaction, respectively. To obtain the optimal set of λ-values, we used the available script at https://gitlab.com/KomBioMol/converge_lambdas.^34^ The optimal set of 30, 30, and 20 windows of λ-values were selected for the mRNA-40S, mRNA-40S-NSP1, and mRNA, respectively. Thus, a total of 80 windows were used for alchemical calculations of free energy. These windows are the same for the full and truncated models. For each window, simulations were run for 1000 ns to ensure that the complexes reached equilibrium. Free energy changes were estimated using the Bennett acceptance ratio.^35^ The binding free energy was then calculated from the thermodynamics cycle (Figure S4).

The CG MARTINI force field allows long-term simulations of large systems by reducing the number of degrees of freedom compared to all-atom models. However, one limitation of the MARTINI model is that it uses an elastic network model, which may introduce artificial stiffness that could affect the free energy calculations. This is an important issue that requires further study, but despite the limitation mentioned here, the free energy estimates obtained with the CG MARTINI model agree reasonably well with experimental results obtained in several previous cases.^28c,36^ From this point of view, our results should be considered as a rough estimate and carefully compared with the SMD and experimental results.

## 3 Results and Discussions

### 3.1 Binding Affinity of mRNA to 40S Ribosome with and without SARS-CoV-2 NSP1: SMD Simulations

**Figure 2:**
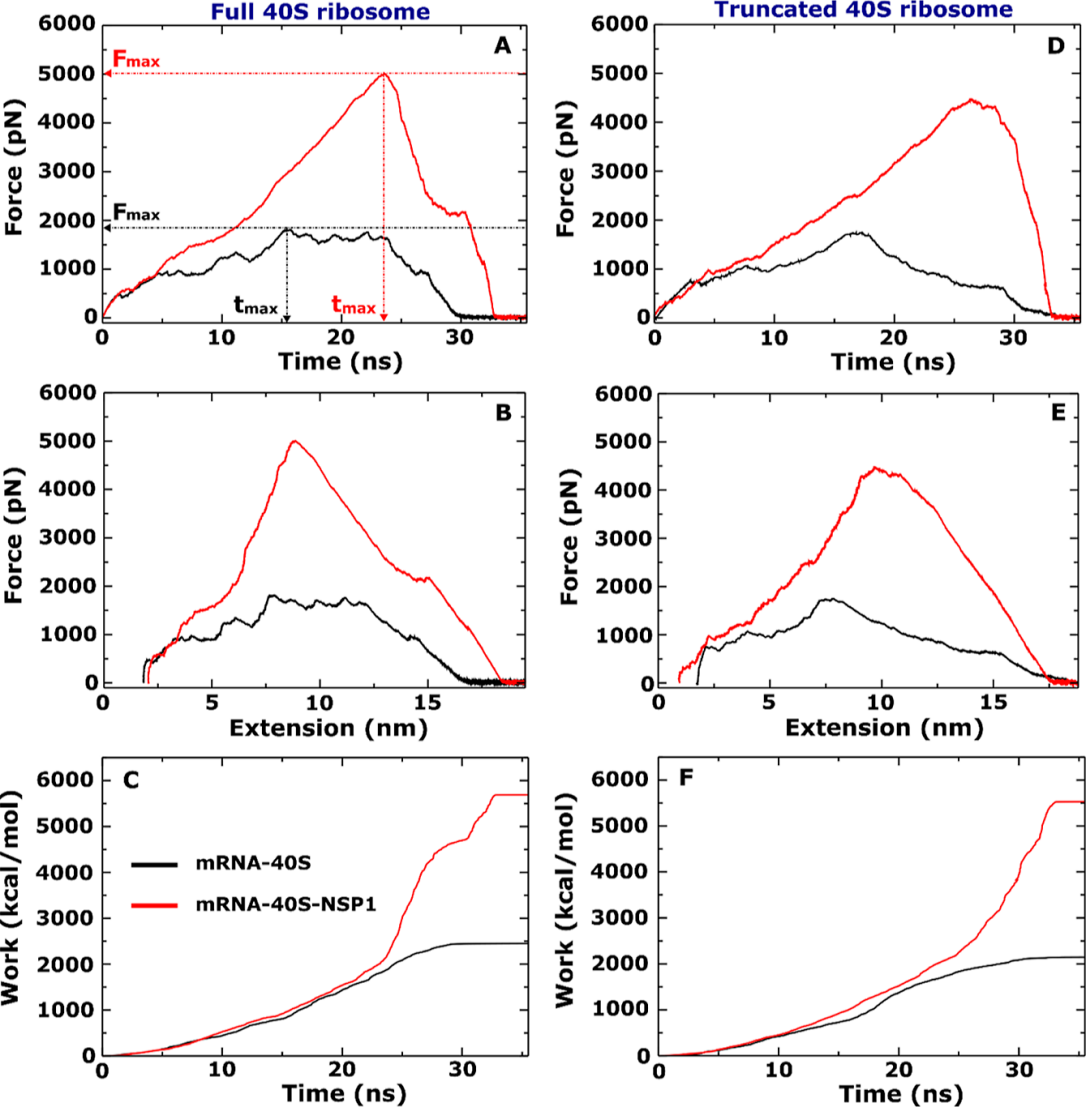
(A,D) Time-dependent behavior of force, (B,E) extension-dependent behavior of force, and (C,F) time-dependent behavior of work profiles of the mRNA-40S (black) and mRNA-40S-NSP1 (red) complexes for the cases of the full 40S ribosome and the truncated version of the 40S ribosome. The results were averaged over 10 independent SMD runs.

Details and setup of SMD simulations are described in SI (Figure S3). The ten most representative structures obtained by clustering snapshots collected during 500 and 1000 ns CMD for the full and truncated 40S ribosome, respectively, were used as starting conformations for the SMD trajectories for the mRNA-40S and mRNA-40S-NSP1 complexes. Figure 2 shows the force, and nonequilibrium work profiles of these complexes, where the result was averaged over 10 independent SMD simulations.

The unbinding pathways can be divided into two distinct parts: before and after reaching the maximum point. For simple systems, such as two interacting proteins without a ribosome, the force–extension profile exhibits linear behavior typical of a spring before rupture.^37^ However, in our case, a nonlinear dependence occurs in all complexes (Figure 2). Beyond the peak, the behavior remains complex, especially in the case of the complete ribosome, where weak peaks appear over large time scales. The mRNA molecule is on the verge of leaving the binding region when the force begins to vanish. Overall, in the presence of NSP1, the complex becomes more rigid, reducing force fluctuations.

Although the first regime in the force-time/extension profile in not linear and several peaks occur in the second regime, the choice of *t*_max_ is not ambiguous, because the main peak (*F*_max_) is clearly higher than other peaks and the dependence of force on time is a single-valued function (Figure 2). For full 40S ribosome, the force–time profile shows that mRNA binds to the 40S-NSP1 (*F*_max_ = 5023.3 ± 232.1 pN) more strongly than to the 40S ribosome (*F*_max_ = 1832.9 ± 127.4 pN). The time to reach the maximum force *t*_max_ increases with increasing *F*_max_ (Figure 2A,B and Table 1).

**Table 1:** Rupture Force (*F*_max_), Rupture Time (*T*_max_), and Non-Equilibrium Work (*W*) Were Averaged Over 10 Independent SMD Trajectories of mRNA-40S and mRNA-40S-NSP1 with the Full and Truncated 40S ribosomes^a^

	mRNA-40S (full 40S ribosome)	mRNA-40S-NSP1 (full 40S ribosome)	mRNA-40S (truncated 40S ribosome)	mRNA-40S-NSP1 (truncated 40S ribosome)
*F*_max_ (pN)	1832.9 ± 127.4	5023.3 ± 232.1	1763.6 ± 103.3	4501.3 ± 227.5
*t*_max_ (ps)	15625.1 ± 1170.5	23666.9 ± 1270.2	17302.7 ± 1208.9	26448.7 ± 1311.3
*W* (kcal/mol)	2401.4 ± 60.8	5688.5 ± 121.2	2147.4 ± 66.2	5526.4 ± 121.3

a The errors represent standard deviations.

Since the nonequilibrium work W is determined for the entire process (eq S2) while *F*_max_ is determined at a single point, W characterizes the binding affinity better than *F*_max_.^38^ Therefore, we also present the results obtained for *W*. Initially, *W* showed an increase as the extended molecule moved out of the binding region, eventually reaching a stable value when the interaction of the mRNA with 40S or 40S-NSP1 disappeared (Figure 2C). In other words, the nonequilibrium work increased until the mRNA separated from the 40S ribosome entry tunnel and became saturated. By defining the work done by mRNA upon exiting the ribosome as the saturation value at the end of the simulation, we obtained *W* = 5688.5 ± 121.2 and 2401.4 ± 60.8 kcal/mol for mRNA-40S-NSP1 and mRNA-40S, respectively (Table 1). Thus the results obtained for both *F*_max_ and *W* indicate that NSP1 increases the binding affinity of mRNA to the ribosome, which interferes with the translation process.

For the truncated 40S ribosome, before rupture the force–extension relationship is not linear, as is the case for the full ribosome (Figure 2D,E), but the overall force–extension/time profile is less complex, likely due to fewer residues interacting with the mRNA. To detach mRNA from the binding region of the 40S-NSP1 complex, a much higher force is required (*F*_max_ = 4501.3 ± 227.5 pN) compared to the case of 40S (*F*_max_ = 1763.6 ± 103.3 pN) (Table 1). For mRNA-40S, the full and truncated ribosome models provide the same *F*_max_, while for mRNA-40S-NSP1 the truncated version gives a slightly lower value within the error bars. The nonequilibrium work shows a further difference in binding affinity caused by NSP1 (Figure 2F), for mRNA-40S-NSP1 *W* = 5526.4 ± 121.3 kcal/mol, and for mRNA-40S, *W* = 2147.4 ± 66.2 kcal/mol (Table 1). Interestingly, *W* is the same for full and truncated ribosomes, both for complexes with and without NSP1. Thus, along with the results obtained for *F*_max_, this result suggests that the truncated ribosome model reasonably predicts relative binding affinities of mRNA, highlighting the increased stability of mRNA-40S-NSP1 compared to mRNA-40S, which is also in good agreement with the experiments on inhibition of mRNA translation by NSP1.^12,16^ Since the relatively small truncated system is easy to equilibrate, this result can be seen as further confirmation of the fact that large complete ribosome models were equilibrated in our simulations.

### 3.2 SARS-CoV-2 NSP1 Binding to 40S Ribosome Reduces the Electrostatic and vdW Interaction Energies between mRNA and 40S Ribosome

van der Waals (Δ*E*_vdW_), electrostatic (Δ*E*_elec_), and total (Δ*E*_total_ = Δ*E*_elec_ + Δ*E*_vdW_) interaction energies averaged over 10 independent SMD runs are shown as a function of simulation time for both mRNA-40S and for mRNA- 40S-NSP1 complexes in full and truncated 40S ribosome models. Δ*E*_vdW_ is negative in the bound state, then reaches 0 kcal/mol in the unbound state for both complexes (Figure S5A,D). In contrast, Δ*E*_elec_ is positive in the bound and unbound states (Figure S5B,E). Clearly, Δ*E*_elec_ is much larger than Δ*E*_vdW_ for the mRNA-40S and mRNA-40S-NSP1 complexes, resulting in Δ*E*_total_ > 0 (Figure S5C,F).

In the full 40S ribosome, the energy of the bound state (*t* < *t*_max_) was determined by averaging over the time interval [0, *t*_max_]. Then Δ*E*_elec_ = 105814.4 ± 301.7 and 125425.2 ± 313.7 kcal/mol, Δ*E*_vdW_ = −308.2 ± 4.2 and −177.6 ± 4.1 kcal/mol, and Δ*E*_total_ = 105506.2 ± 305.9 and 125247.6 ± 317.8 kcal/mol for the mRNA-40S-NSP1 and the mRNA-40S complexes, respectively (Table 2). For the truncated 40S ribosome model, the following energy values were obtained for the mRNA-40S-NSP1 and the mRNA-40S complexes: Δ*E*_elec_ = 57929.2 ± 216.5 and 71538.9 ± 225.4 kcal/mol, Δ*E*_vdW_ = −305.3 ± 3.7 and −269.6 ± 4.4 kcal/mol, and Δ*E*_total_ = 57623.9 ± 220.2 and 71269.3 ± 229.8 kcal/mol, respectively (Table 2). Although Δ*E*_vdW_, Δ*E*_elec_, and Δ*E*_total_ differ for full and truncated 40S ribosomes, these results indicate that the interaction between mRNA and 40S ribosome is reduced by SARS-CoV-2 NSP1 binding.

**Table 2:** Non-Bonded Interaction Energies (kcal/mol) of the mRNA-40S and mRNA-40S-NSP1 with Both the Full and the Truncated 40S Ribosomes^a^

	mRNA-40S (full 40S ribosome)	mRNA-40S-NSP1 (full 40S ribosome)	mRNA-40S (truncated 40S ribosome)	mRNA-40S-NSP1 (truncated 40S ribosome)
Δ*E*_elec_	125425.2 ± 313.7	105814.4 ± 301.7	71538.9 ± 225.4	57929.2 ± 216.5
Δ*E*_vdW_	–177.6 ± 4.1	–308.2 ± 4.2	–269.6 ± 4.4	–305.3 ± 3.7
Δ*E*_total_	125247.6 ± 317.8	105506.2 ± 305.9	71269.3 ± 229.8	57623.9 ± 220.2

a The results were obtained for a [0-*t*_max_] time window and averaged over 10 SMD trajectories. The errors represent standard deviations.

Thus, for both complexes, the electrostatic interaction predominates over the vdW interaction. The positive value of Δ*E*_total_ is due to repulsion between negatively charged 40S ribosome (−1215e), mRNA (−21e), and SARS-CoV-2 NSP1 (−3e) (Table S1). Our result also shows that SARS-CoV-2 NSP1 binding reduces the interaction between mRNA and 40S ribosome, making the mRNA-40S complex more stable.

Note that the AMBER99SB force field we use is a nonpolarizable force field that neglects charge regulation effects. This may lead to inaccurate predictions of electrostatic interactions of mRNA with surrounding molecules. Therefore, we should be cautious in concluding that Coulomb electrostatic interactions play a more important role than van der Waals interactions for mRNA stability.

### 3.3 Water Molecules Stabilize the Systems

Since the total interaction energy Δ*E*_total_ obtained in the previous section is positive for both complexes, an important question emerges is whether these complexes are stable? To answer this question we will take into account water molecules. Again, Δ*E*_total_ was calculated by averaging over 10 SMD trajectories in the time window [0, *t*_max_] for the full 40S ribosome. We obtained the total energy of −288942.4 ± 212.5, and −311478.3 ± 267.3 kcal/mol for the mRNA-40S and the mRNA-40S-NSP1, respectively (Table S2), which implies that these complexes are stabilized by water molecules.

### 3.4 Important SARS-CoV-2 NSP1 Residues

**Figure 3:**
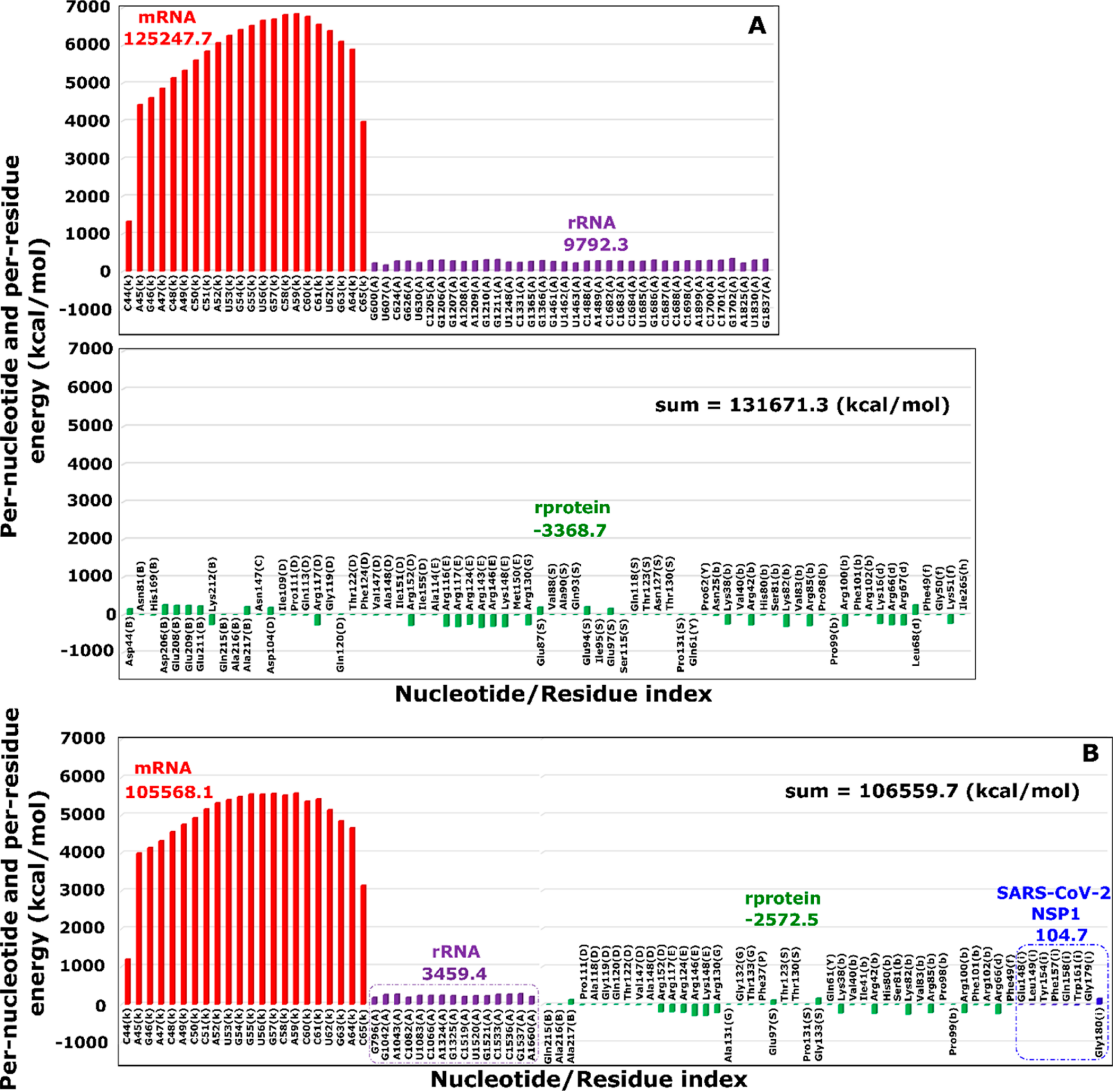
Interaction energy (electrostatic and vdW) per-nucleotide and per-residue at the binding regions of mRNA to (A) 40S ribosome and (B) 40S-NSP1. The numbers next to the profiles refer to the total energy (sum over all interacting residues or nucleotides) measured in kcal/mol. For example, for mRNA in (A) the sum over all nucleotides and residues in the binding site is 131671.3 kcal/mol, while in (B) it is only 106559.7 kcal/mol. The results were averaged over 10 independent SMD runs of the full 40S ribosome.

The energy per nucleotide of mRNA and rRNA, as well as the energy per residue of rprotein and SARS-CoV-2 NSP1 are shown in Figure 3 for mRNA-40S and mRNA-40S-NSP1 complexes. They were obtained by averaging over 10 SMD trajectories in the [0, *t*_max_] time window only for the full 40S ribosome case. This took into account the interaction of mRNA with all rproteins, rRNA and NSP1 of SARS-CoV-2 for the mRNA-40S and mRNA-40S-NSP1 complexes. Clearly, the energy of mRNA per nucleotide is much higher than that of rRNA per nucleotide, rprotein per residue, and SARS-CoV-2 NSP1 per residue. It is important to note that the total energy of nucleotides and residues of mRNA-40S-NSP1 (106559.7 kcal/mol) is significantly less than that of mRNA-40S (131671.3 kcal/mol) (Figure 3A,B). This result is consistent with the result obtained for the entire system, including the binding region, that SARS-CoV-2 NSP1 reduces the interaction between mRNA and the 40S ribosome upon binding to the mRNA channel.

Moreover, the contribution of each SARS-CoV-2 NSP1 residue at the binding region to the binding energy is Glu148 = −4.4, Leu149 = −3.9, Tyr154 = −7.5, Phe157 = −9.4, Gln158 = −8.4, Trp161 = −20.5, Gly179 = −1.6, and Gly180 = 160.6 kcal/mol (Figure 3B). Since the interaction energy of Glu148, Leu149, Tyr154, Phe157, Gln158, Trp161 and Gly179 is negative, these residues stabilize the system, whereas with positive interaction energy only Gly180 makes the complex less stable. Although the SARS-CoV-2 NSP1 total energy of interaction with mRNA is positive (104.7 kcal/mol), its presence makes the complex more stable by reducing the interaction energy of mRNA with rRNA and rprotein. Taken together, mRNA translation at the 40S ribosome of the host immune system is controlled by electrostatic interactions and can be stalled by SARS-CoV-2 NSP1. SARS-CoV-2 NSP1 residues Glu148, Leu149, Tyr154, Phe157, Gln158, Trp161, Gly179, and Gly180 play a key role as they are at the interface with mRNA.

### 3.5 Binding Free Energy of mRNA to the 40S Ribosome with and without SARS-CoV-2 NSP1: Alchemical Simulations

Figure S6 displays the time dependence of rmsd of mRNA, mRNA-40S, and mRNA-40S-NSP1 at λ = 0 for both the full 40S ribosome and the truncated 40S ribosome cases. This plot shows that these systems achieved equilibrium after approximately 200 ns. As a result, we proceeded to calculate the binding free energy of mRNA to the 40S ribosome and 40S-NSP1 using two different time windows: [200–800 ns] and [200–1000 ns] (Table 3). It is worth noting that the results obtained in these two time windows are similar within the margin of error, indicating that the data were indeed equilibrated. Therefore, we decided to base our analysis on the results obtained from the [200–1000 ns] time window.

**Table 3:** Binding Free Energies (kcal/mol) of the mRNA-40S and mRNA-40S-NSP1 Complexes with Both the Full 40S Ribosome and the Truncated 40S Ribosome^a^

		full 40S ribosome		truncated 40S ribosome	
		mRNA-40S	mRNA-40S-NSP1	mRNA-40S	mRNA-40S-NSP1
Δ*G*_bind,_ Experiment^39^	–10.7 ± 0.1	N/A	N/A	N/A	
Δ*G*_bind_^ALC^, Our simulation	200–800 ns	–12.7 ± 1.2	–35.8 ± 2.1	–8.3 ± 1.7	–27.9 ± 3.1
	200–1000 ns	–13.1 ± 1.1	–37.1 ± 2.2	–8.6 ± 1.2	–28.2 ± 2.6

a The results were obtained using alchemical free energy calculations and the MARTINI CG model.

For the full 40S ribosome, the binding free energy of mRNA-40S, denoted as Δ*G*_bind_^ALC^ = −13.1 ± 1.1 kcal/mol, which is very close to the experimental value of −10.7 ± 0.1 kcal/mol.^39^ In contrast, in the presence of NSP1, the binding free energy of mRNA-40S-NSP1 is reduced to Δ*G*_bind_^ALC^ = −37.1 ± 2.2 kcal/mol. The binding affinity increases approximately 3-fold at a ratio of R = Δ*G*_bind_^ALC^(mRNA-40S-NSP1)/Δ*G*_bind_^ALC^(mRNA-40S) = −37.1/–13.1 = 2.8. Thus, consistent with the SMD results, NSP1 strongly increases the binding affinity of mRNA to the entry channel, stopping translation and hence the protein synthesis process.^12,16^

For the truncated 40S ribosome, the binding free energy of mRNA-40S the binding free energy (Δ*G*_bind_^ALC^ = −8.6 ± 1.2 kcal/mol) is higher than that of mRNA-40S-NSP1 (Δ*G*_bind_^ALC^ = −28.2 ± 2.6 kcal/mol) (Table 3). The fact that the absolute value of Δ*G*_bind_^ALC^ of a truncated ribosome is lower than a full ribosome is reasonable since the smaller system must be less stable than larger one. Nevertheless our results obtained for the truncated complexes also support the main conclusion that NSP1 suppresses mRNA translation by increasing binding affinity (*R* = Δ*G*_bind_^ALC^(mRNA-40S-NSP1Δ*G*_bind_^ALC^)/(mRNA-40S) = −28.2/–8.6 = 3.3). This *R* ratio is higher than in the case of a complete ribosome.

Since nonequilibrium work is a good measure of binding affinity, *R* can be defined as *R* = *W*(mRNA-40S-NSP1)/*W*(mRNA-40S). Using the SMD data shown in Table 1, we obtain *R* = 2.4 and 2.6 for the full and truncated complexes, respectively. These values are not far from 2.8 obtained from the binding free energies of the full ribosome complexes. Moreover, both SMD and alchemical simulations yield *R* of full ribosome complexes lower than the truncated case.

## 4 Conclusion

In conclusion, our study employed a combination of SMD and alchemical simulations to investigate the association of mRNA with the 40S ribosome, both in the absence and presence of SARS-CoV-2 NSP1. Our all-atom SMD results clearly demonstrate that mRNA exhibits a much stronger binding affinity to the 40S-NSP1 complex than to the 40S ribosome alone. This observation aligns with the results obtained from the binding free energy calculations using CG alchemical simulations. Therefore, it can be inferred that the mRNA-40S complex is relatively less stable when compared to the mRNA-40S-NSP1 complex. Our findings are in excellent agreement with experimental data from previous studies.^12,16^ It is shown that the mRNA translation process is primarily driven by the electrostatic interactions between mRNA and the 40S ribosome. Upon entering host cells, SARS-CoV-2 NSP1 has the potential to bind to the 40S ribosome, thereby inhibiting the translation process. Our analysis identified key SARS-CoV-2 NSP1 residues, including Glu148, Leu149, Tyr154, Phe157, Gln158, Trp161, Gly179, and Gly180, at the interface with mRNA, which play a crucial role in triggering translational arrest of the host immune system.

## Supporting Information Available

A brief overview of the methods used to estimate binding affinity using molecular dynamics simulations is provided, as well as a detailed description of conventional and SMDs simulations. Figure S1: the scheme describes all MD simulations in this work. Figure S2: rmsd as a function of simulation time of mRNA-40S, and mRNA-40S-NSP1 complexes with the full and truncated 40S ribosome. Figure S3: initial and final conformations from SMDs simulation of the extraction of mRNA from 40S ribosomal subunit and SARS-CoV-2 NSP1 for both full and truncated 40S ribosome. Figure S4: an example of a thermodynamics cycle to calculate binding free energy between mRNA and the 40S-NSP1 using alchemical simulation. Figure S5: time dependence of vdW, electrostatic, and total interaction energy of mRNA-40S and mRNA-40S-NSP1 for full and truncated 40S ribosomes. Figure S6: rmsd as a function of simulation time of only mRNA, mRNA-40S, mRNA-40S-NSP1 at λ = 0 in the alchemical free energy calculations using the MARTINI CG model. Table S1: Total charge of the 40S ribosome, SARS-CoV-2 NSP1 and mRNA. Table S2: total nonbonded energy of the mRNA-40S and mRNA-40S-NSP1 complexes with water and ions (PDF)
