# Supplementary material for: Binding of SARS-CoV-2 Nonstructural Protein 1 to 40S Ribosome Inhibits mRNA Translation
Source: J Phys Chem B. 2024 Jul 15;128(29):7033–42. doi: 10.1021/acs.jpcb.4c01391 (PMC11284778; doi:10.1021/acs.jpcb.4c01391)
Supplement: Supplementary file 1 — jp4c01391_si_001.pdf [file jp4c01391_si_001.pdf]

Supporting Information

Binding of SARS-CoV-2 Non-structural Protein 1 to 40S Ribosome Inhibits mRNA Translation

Hung Nguyen[1], **Hoang Linh Nguyen**[3,4], and **Mai Suan Li**[1,2,*]

[1]*Institute of Physics, Polish Academy of Sciences, al. Lotnikow 32/46, 02-668, Warsaw, Poland.*

[2]*Life Science Lab, Institute for Computational Science and Technology, Quang Trung Software City, Tan Chanh Hiep Ward, District 12, Ho Chi Minh City 729110, Vietnam.*

[3]*Institute of Fundamental and Applied Sciences, Duy Tan University, Ho Chi Minh City 700000, Vietnam.*

[4]*Faculty of Environmental and Natural Sciences, Duy Tan University, Da Nang City 550000, Vietnam.*

[*]Email: *masli@ifpan.edu.pl*

Table of Content

Supporting Methods..........S2

Estimation of Binding Affinity Using Molecular Dynamics Simulations..........S2

Conventional and Steered Molecular Dynamics Simulations..........S2

Supporting Figures..........S5

Figure S1..........S5

Figure S2..........S6

Figure S3..........S7

Figure S4..........S8

Figure S5..........S9

Figure S6..........S10

Supporting Tables..........S11

Table S1..........S11

Table S2..........S11

Supporting References..........S12

Supporting Methods

Estimation of binding affinity using molecular dynamics simulations

Several methods have been proposed to evaluate the binding free energy of protein-ligand, protein-protein, protein-DNA/RNA complexes [1], such as thermodynamics integration (TI) [2], free energy perturbation (FEP) [3], molecular mechanics with Poisson-Boltzmann or generalized Born and surface area (MM-PBSA and MM-GBSA) [4], linear interaction energy (LIE) [5], docking molecular dynamics [6], and steered molecular dynamics (SMD) [7].

The alchemical free energy calculations referred to as TI or FEP, which is based on molecular dynamics (MD) simulation in explicit solvent, is known to be the most accurate method due to its high level of theoretical rigor, and is also accessible within existing computational powers for relatively large systems. This method is based on the non-physical thermodynamics cycle, where the binding free energy is calculated as the sum of multiple steps during which a ligand/protein/DNA/RNA is “inserted” or “removed” from different states, including bound and unbound states [8]. The alchemical free energy calculation has been successful in determining free energy difference in many situations such as the partition of a compound between different media, the binding affinity of protein-ligand, protein-protein, protein-DNA/RNA complexes with mutations at their interface [9]. The free energy difference can be calculated using alchemical routes of relative or absolute binding free energies [10].

In this study, we conducted all-atom SMD simulations and a set of alchemical free energy calculations using the MARTINI coarse-grained model to evaluate the binding affinity of mRNA to the 40S and 40S-NSP1 complexes.

Conventional and steered molecular dynamics simulations

Before starting SMD and alchemical simulations, we performed a 1000 ns coarse-grained molecular dynamics (CGMD) simulation for both the mRNA-40S and mRNA-40S-NSP1 complexes. The final frame of the CGMD simulations was then converted to an all-atom structure using the backward script [11]. The resulting all-atom structure was used to run all-atom conventional molecular dynamics (CMD) simulations for 500 ns for the mRNA-40S and mRNA-40S-NSP1 complexes, which generate initial configurations for SMD and alchemical simulations.

In detail, for CGMD simulations, these complexes were placed in a dodecahedron box with a distance between the solute and the box of 1.2 nm. The energy of the system was first minimized, and then followed by a 5 ns NVT and NPT simulations. A 1000 ns CGMD

simulation was then performed for the systems using MARTINI force field [12]. The root mean square deviation (RMSD) versus time from the CGMD simulation is shown in Figure S2A. Obviously, the two complexes have reached equilibrium as RMSD remains below 3 Å, but it remains unclear whether the CG structure is stable in all-atom models.

Therefore, the last snapshot was converted to the all-atom structure, which was used as the initial structure for the all-atom CMD simulation. In this simulation, the complexes were placed in a dodecahedron box with a distance of 1.2 nm between the solute and the box. The system energy was then minimized using the steepest descent algorithm followed by a short 3 ns CMD simulation in the NVT and NPT ensembles. Finally, a 500 ns production CMD simulation was conducted using the leap-frog algorithm [13] for these systems. As can be seen from the RMSD time dependence (Figure S2B), the complexes are also stable in the all-atom models after about 200 ns. Applying the clustering analysis to the collected snapshots from a 500 ns all-atom CMD run, we obtained 10 representative structures that will be used as the initial structure to run 10 independent SMD simulations. The most populated structure obtained by clustering snapshots collected in equilibrium from the 500 ns CMD simulations for the mRNA-40S and mRNA-40S-NSP1 complexes were used to perform the alchemical simulations using MARTINI coarse-grained model.

Additionally, we utilized the most populated snapshot obtained from the clustering analysis of 500 ns CMD runs, which served as the basis for truncating the mRNA-40S and mRNA-40S-NSP1 complexes at the binding regions of mRNA to the 40S and 40S-NSP1 (Figure 1C). Subsequently, we truncated the mRNA-40S and mRNA-40S-NSP1 systems and performed all-atom conventional molecular dynamics simulations, extending the simulation time to 1000 ns. For this case, the carbon alpha (C_α) atoms of ribosomal protein and phosphate (P) atoms of ribosomal RNA at the truncated surfaces were restrained during the simulations. The RMSD time dependence analysis (Figure S2C) demonstrates that the complexes also reached a stable stage during the simulation. Following this, we conducted a clustering analysis to select 10 representative snapshots. These 10 structures were then employed as the initial configurations for 10 independent SMD simulations. Additionally, The most populated structure from clustering snapshots of 1000 ns CMD simulations for the truncated mRNA-40S and mRNA-40S-NSP1 complexes were also used to conduct the alchemical simulations using MARTINI coarse-grained model. The purpose is to compare the results obtained from the truncated complexes with those of the full 40S complexes, to assess their similarity and make sure that our structures were equilibrated.

SMD simulations [7] were carried out to pull mRNA from the ribosomal 40S exit tunnel for cases with and without SARS-CoV-2 NSP1 for the full and truncated 40S complexes. Pulling speeds *v* = 0.5 nm/ns was used resulting in a simulation time of ~35 ns. This value of *v* is about ten orders of magnitude larger than in the experiment, but as shown in previous works [14], this choice does not influence relative binding affinities, *i.e.*, it can be used to discern strong binders from weak ones. A rectangular box with the dimension of 32×25×55 nm^3 was used for all systems of the full 40S system, and 15×17×40 nm^3 was applied to the truncated 40S systems. These complexes were immersed in 0.15 M sodium chloride solution and counter ions were added to neutralize the system. To pull the mRNA out of the exit tunnel, an external force is applied to the dummy atom connected to the 5'-mRNA (O5' atom), through a spring with a stiffness of k. The complexes were rotated so that the exit direction was parallel to the z-axis, which is along the mRNA entry channel (Figure S3).

The force experienced by a stretched molecule is calculated as follows:

$$F = k(\Delta z - vt) \quad (S1)$$

where *k* is the stiffness of the spring, v is the pulling speed, Δz is the displacement of the atom connected to the spring in the direction of pulling. The spring constant *k* was set to 600 kJ/(mol.nm^2) (≈ 1020 pN/nm), which is a typical value used in atomic force microscopy (AFM) experiments [15].

Using the force-displacement profile obtained from SMD simulations, the non-equilibrium work (W) can be calculated using the trapezoidal rule:

$$W = \int F dz = \sum_{i=1}^{N} \frac{F_{i+1} + F_i}{2} (z_{i+1} - z_i) \quad (S2)$$

where *N* is the number of simulation steps, F_i and z_i are the force defined by Eq. S1 and the position at step *i*, respectively.

The CMD and SMD simulations were performed using the AMBER99SB force field [16] implemented in the GROMACS 2022 package [17]. The v-rescale [18] and Parrinello-Rahman [19] algorithms were used to maintain the temperature at 300 K and the isotropic pressure at 1 bar, respectively. The water model TIP3P [20] was used for all systems. Bond lengths were constrained by the linear constraint solver (LINCS) algorithm [21] allowing a time step of 2 fs. The electrostatic and van der Waals interactions were calculated using a cutoff of 1.2 nm, and the non-bonded interaction pair-list was updated every 10 fs. The Particle Mesh Ewald algorithm [22] was used to treat long-range electrostatic interactions. Periodic boundary conditions were applied in all directions.

Supporting Figures

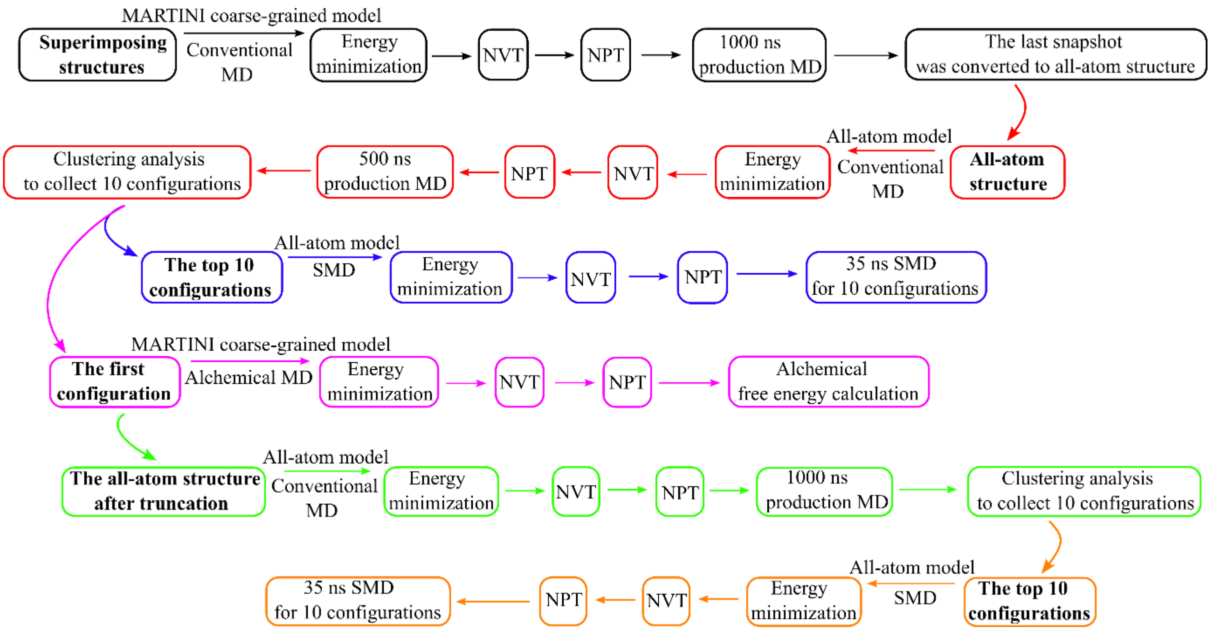

Figure S1: The scheme describes all MD simulations in this work, including MARTINI coarse-grained MD (CGMD, black), all-atom conventional MD for the full 40S ribosome (CMD, red), all-atom SMD for the full 40S ribosome (blue), alchemical MD using the MARTINI model (magenta), all-atom conventional MD for the truncated 40S ribosome (green), and all-atom SMD for the truncated 40S ribosome (orange).

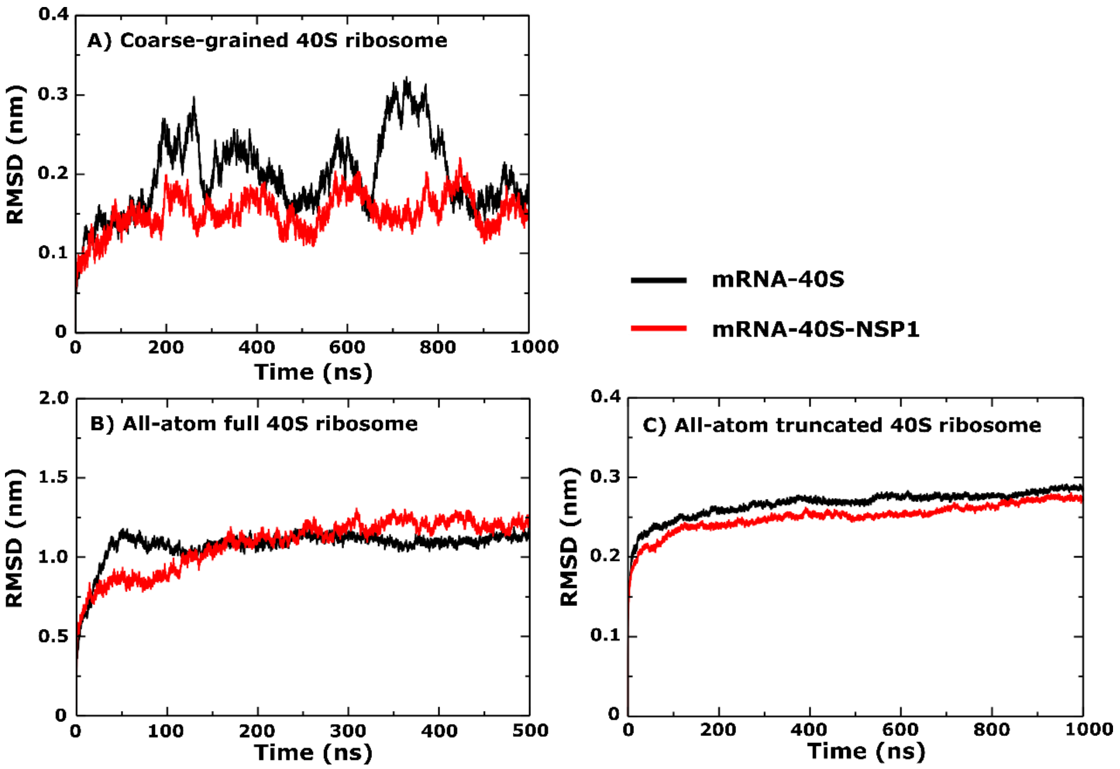

Figure S2: Root mean square deviation (RMSD) as a function of simulation time of mRNA-40S (back), and mRNA-40S-NSP1 (red) complexes for A) Coarse-grained conventional MD, B) all-atom CMD of the full 40S ribosome and C) all-atom CMD of the truncated 40S ribosome.

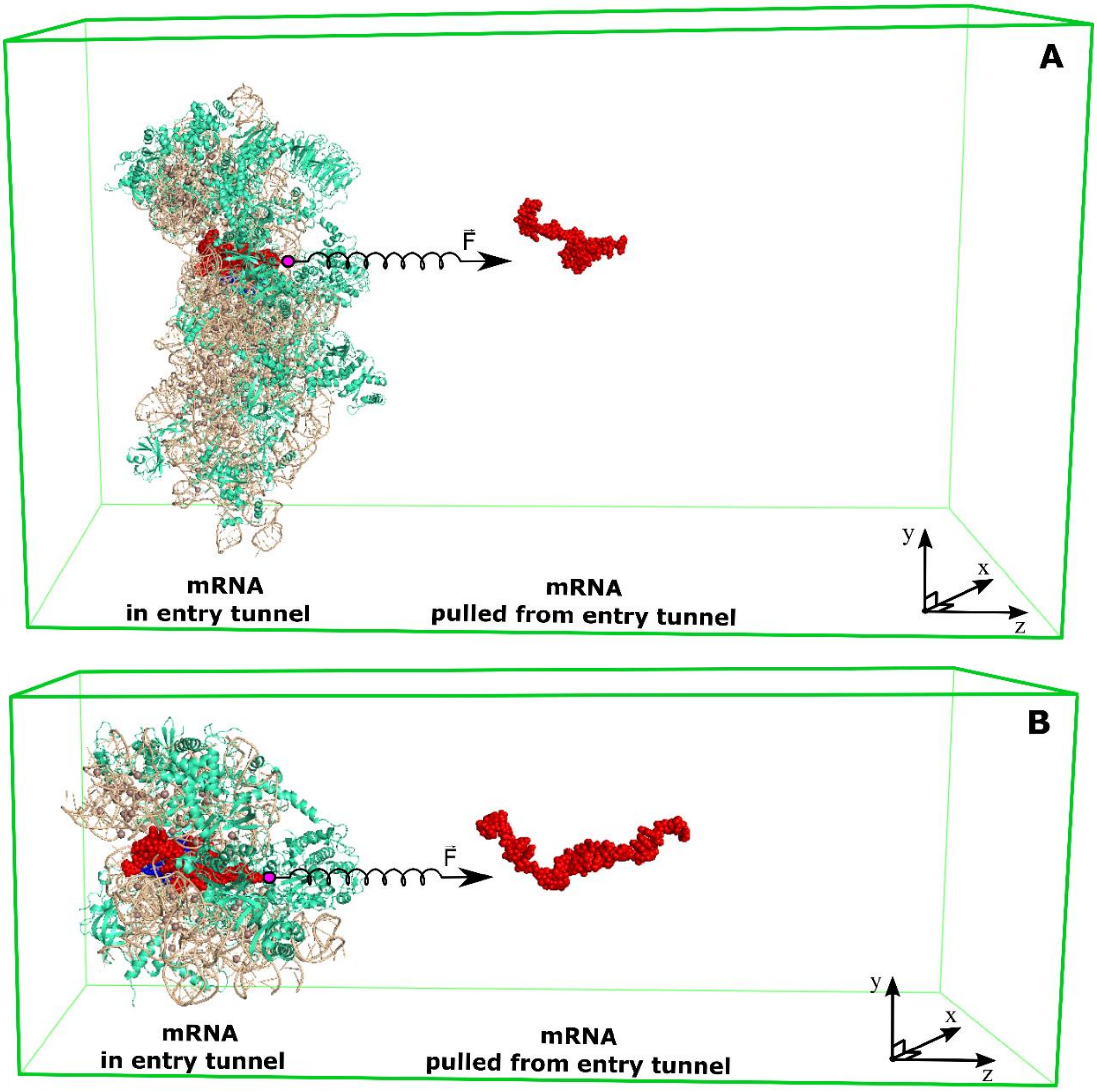

Figure S3: Initial (left) and final (right) conformations from steered molecular dynamics simulation of the extraction of mRNA (red) from 40S ribosomal subunit (green-cyan: ribosomal protein (rprotein), and wheat: ribosomal RNA (rRNA)) and SARS-CoV-2 NSP1 (blue) for A) the full 40S ribosome, and B) the truncated 40S ribosome.

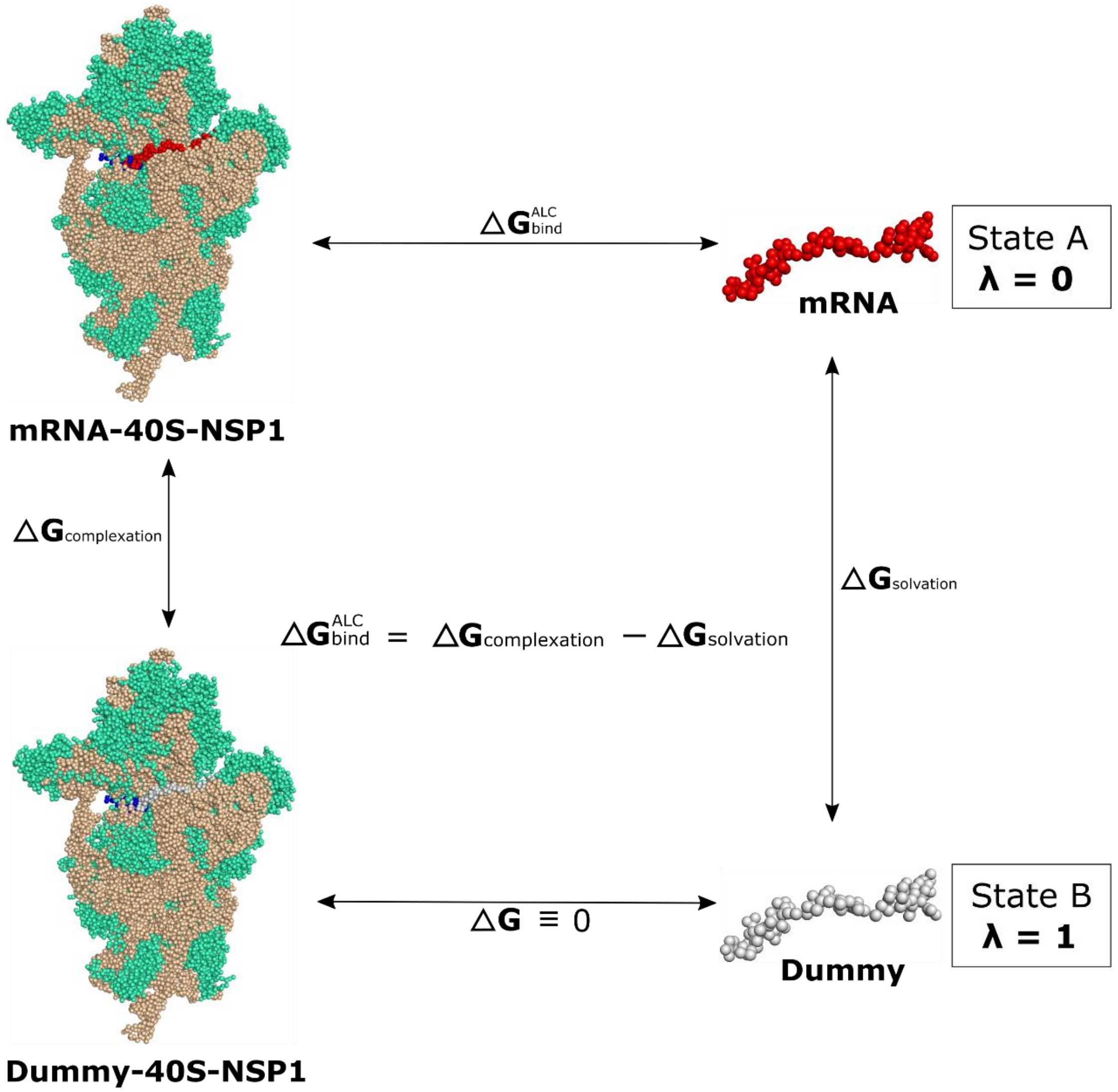

Figure S4: An example of a thermodynamics cycle to calculate binding free energy between mRNA and the 40S-NSP1 using alchemical simulation. State A ($\lambda = 0$) describes full interaction between mRNA and 40S-NSP1 while state B ($\lambda = 1$) presents mRNA (dummy) of no interaction with 40S-NSP1. These structures are displayed in rRNA (wheat), rprotein (green-cyan), mRNA dummy (gray), mRNA (red), and SARS-CoV-2 NSP1 (blue). Alchemical free energy calculations are used in the MARTINI coarse-grained model.

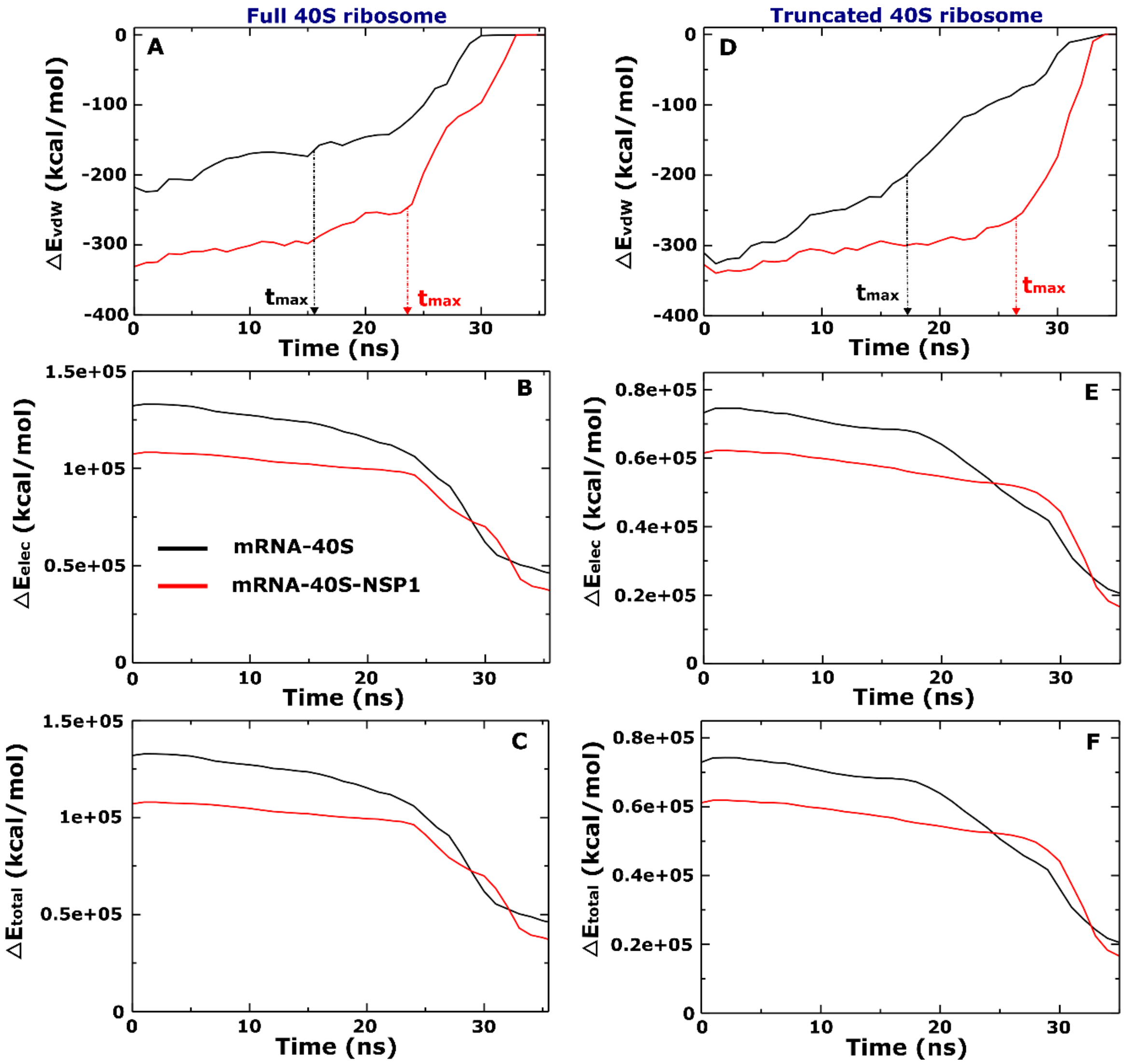

Figure S5: Time dependence of (A, D) vdW, (B, E) electrostatic, and (C, F) total interaction energy of mRNA-40S (black) and mRNA-40S-NSP1 (red) for full and truncated models 40S ribosomes. The results were averaged over 10 independent SMD runs.

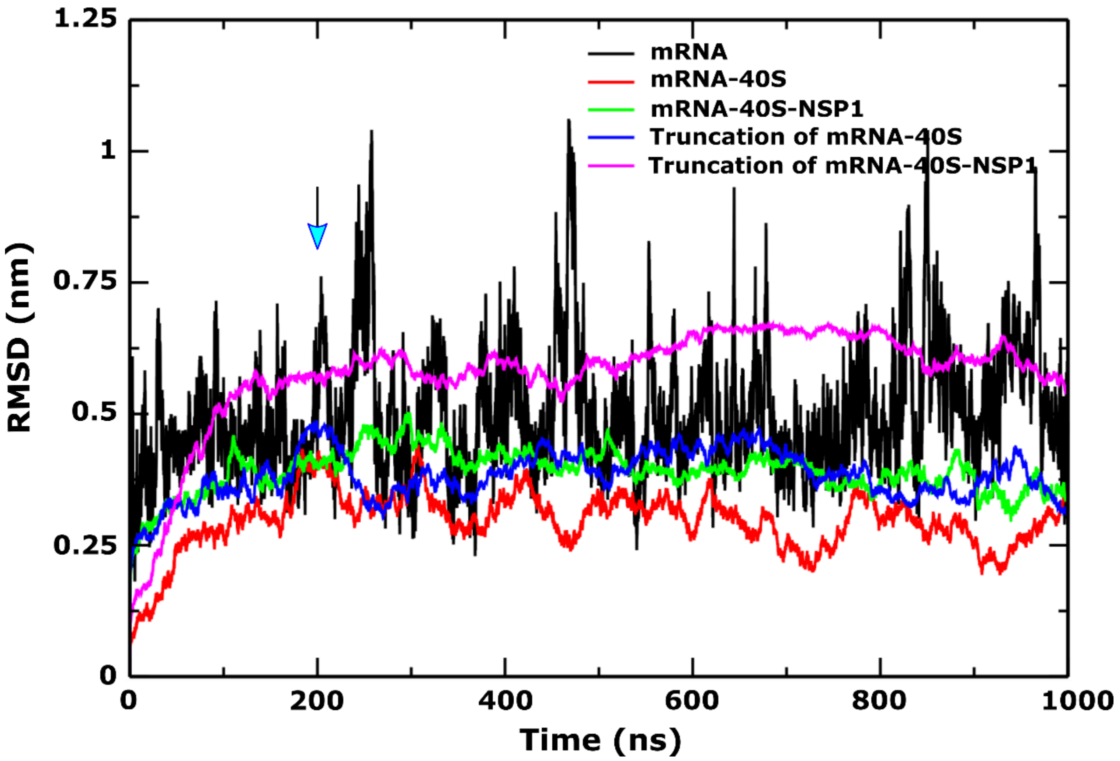

Figure S6: Root-mean-square deviation (RMSD) as a function of simulation time of only mRNA (black), mRNA-40S (red), mRNA-40S-NSP1 (green), truncation of mRNA-40S (blue), and truncation of mRNA-40S-NSP1 (magenta) at $\lambda = 0$ in the alchemical free energy calculations using the MARTINI coarse-grained model. The arrow indicates the time (200 ns) when the system reaches equilibrium.

Supporting Tables

Table S1: Total charge of the 40S ribosome, SARS-CoV-2 NSP1 and mRNA.

Structure	Total charge (e)
40S ribosome	-1215
SARS-CoV-2 NSP1	-3
mRNA	-21

Table S2: Total non-bonded energy of the mRNA-40S and mRNA-40S-NSP1 complexes with water and ions (kcal/mol). The results were averaged over 10 independent SMD runs for the time window [0, t_{max}] for full 40S ribosome and mRNA with and without SARS-CoV-2 NSP1.

Complex	Total non-bonded energy
mRNA-40S with water and ions	-288942.4±212.5
mRNA-40S-NSP1 with water and ions	-311478.3±267.3

Supporting References

1. Marshall, G. R., Computer-aided Drug Design. *Annu. Rev. Pharmacol. Toxicol.* **1987,** *27*, 193-213.
2. Kumar, J.; Dey, T. K.; Sinha, S. K., Semiclassical Statistical Mechanics of Hard-body Fluid Mixtures. *J. Chem. Phys.* **2005,** *122* (22), 224504.
3. Zwanzic, R. W., High-temperature Equation of State by A Perturbation Method .I. Nonpolar Gases. *J. Chem. Phys.* **1954,** *22*, 1420.
4. Srinivasan, J.; Miller, J.; Kollman, P. A.; Case, D. A., Continuum Solvent Studies of the Stability of RNA Hairpin Loops and Helices. *J. Biomol. Struct. Dyn.* **1998,** *16* (3), 671-82.
5. Aqvist, J.; Medina, C.; Samuelsson, J. E., A New Method for Predicting Binding Affinity in Computer-aided Drug Design. *Protein Eng.* **1994,** *7* (3), 385-91.
6. Van Nguyen, H.; Nguyen, H. T.; Le, L. T., Investigation of the Free Energy Profiles of Amantadine and Rimantadine in the AM2 Binding Pocket. *Eur. Biophys. J.* **2016,** *45* (1), 63-70.
7. (a) Nguyen, H.; Do, N.; Phan, T.; Pham, T., Steered Molecular Dynamics for Investigating the Interactions Between Insulin Receptor Tyrosine Kinase (IRK) and Variants of Protein Tyrosine Phosphatase 1B (PTP1B). *Appl. Biochem. Biotechnol.* **2018,** *184* (2), 401-413; (b) Lu, H.; Schulten, K., Steered Molecular Dynamics Simulations of Force-induced Protein Domain Unfolding. *Proteins* **1999,** *35* (4), 453-63; (c) Pham, T.; Nguyen, H. L.; Phan-Toai, T.; Nguyen, H., Investigation of Binding Affinity between Potential Antiviral Agents and PB2 Protein of Influenza A: Non-equilibrium Molecular Dynamics Simulation Approach. *Int. J. Med. Sci.* **2020,** *17* (13), 2031-2039.
8. (a) Chipot, C., Frontiers in Free-energy Calculations of Biological Systems. *Wiley Interdiscip. Rev. Comput. Mol. Sci.* **2014,** *4*; (b) Frenkel, D.; Smit, B., *Understanding Molecular Simulation: From Algorithms to Applications. 2nd ed*. 1996; Vol. 50; (c) Hansen, N.; van Gunsteren, W. F., Practical Aspects of Free-energy Calculations: A Review. *J. Chem. Theory. Comput.* **2014,** *10* (7), 2632-47.
9. Chodera, J. D.; Mobley, D. L.; Shirts, M. R.; Dixon, R. W.; Branson, K.; Pande, V. S., Alchemical Free Energy Methods for Drug Discovery: Progress and Challenges. *Curr. Opin. Struct. Biol.* **2011,** *21* (2), 150-60.
10. Klimovich, P. V.; Mobley, D. L., A Python Tool to Set Up Relative Free Energy Calculations in GROMACS. *J. Comput. Aided Mol. Des.* **2015,** *29* (11), 1007-14.
11. Wassenaar, T. A.; Pluhackova, K.; Bockmann, R. A.; Marrink, S. J.; Tieleman, D. P., Going Backward: A Flexible Geometric Approach to Reverse Transformation from Coarse Grained to Atomistic Models. *J. Chem. Theory. Comput.* **2014,** *10* (2), 676-90.
12. (a) Monticelli, L.; Kandasamy, S. K.; Periole, X.; Larson, R. G.; Tieleman, D. P.; Marrink, S. J., The MARTINI Coarse-Grained Force Field: Extension to Proteins. *J. Chem. Theory. Comput.* **2008,** *4* (5), 819-34; (b) Uusitalo, J. J.; Ingolfsson, H. I.; Marrink, S. J.; Faustino, I., Martini Coarse-grained Force Field: Extension to RNA. *Biophys. J.* **2017,** *113* (2), 246-256; (c) Uusitalo, J. J.; Ingolfsson, H. I.; Akhshi, P.; Tieleman, D. P.; Marrink, S. J., Martini Coarse-grained Force Field: Extension to DNA. *J. Chem. Theory Comput.* **2015,** *11* (8), 3932-45.
13. Hockney, R. W.; Goel, S. P.; Eastwood, J. W., Quiet High-resolution Computer Models of A Plasma. *J. Comput. Phys.* **1974,** *14* (2), 148-158.
14. (a) Nguyen, H.; Lan, P. D.; Nissley, D. A.; O'Brien, E. P.; Li, M. S., Electrostatic Interactions Explain the Higher Binding Affinity of the CR3022 Antibody for SARS-CoV-2 Than the 4A8 Antibody. *J. Phys. Chem. B* **2021,** *125* (27), 7368-7379; (b) Nguyen, H.; Lan, P.

D.; Nissley, D. A.; O'Brien, E. P.; Li, M. S., Cocktail of REGN Antibodies Binds More Strongly to SARS-CoV-2 Than Its Components, but the Omicron Variant Reduces Its Neutralizing Ability. *J. Phys. Chem. B* **2022,** *126* (15), 2812-2823; (c) Nguyen, H.; Li, M. S., Antibody-nanobody Combination Increases Their Neutralizing Activity Against SARS-CoV-2 and Nanobody H11-H4 Is Effective Against Alpha, Kappa and Delta Variants. *Sci. Rep.* **2022,** *12* (1), 9701.
15. Binnig, G.; Quate, C. F.; Gerber, C., Atomic Force Microscope. *Phys. Rev. Lett.* **1986,** *56* (9), 930-933.
16. Hornak, V.; Abel, R.; Okur, A.; Strockbine, B.; Roitberg, A.; Simmerling, C., Comparison of Multiple Amber Force Fields and Development of Improved Protein Backbone Parameters. *Proteins* **2006,** *65* (3), 712-25.
17. Abraham, M. J.; Murtola, T.; Schulz, R.; Páll, S.; Smith, J. C.; Hess, B.; Lindahl, E., GROMACS: High Performance Molecular Simulations Through Multi-level Parallelism from Laptops to Supercomputers. *SoftwareX* **2015,** *1-2*, 19-25.
18. Bussi, G.; Donadio, D.; Parrinello, M., Canonical Sampling Through Velocity Rescaling. *J. Chem. Phys.* **2007,** *126* (1), 014101.
19. Parrinello, M.; Rahman, A., Polymorphic Transitions in Single Crystals: A New Molecular Dynamics Method. *J. Appl. Phys.* **1981,** *52*, 7182-7190.
20. Jorgensen, W. L.; Jenson, C., Temperature Dependence of TIP3P, SPC, and TIP4P Water from NPT Monte Carlo Simulations: Seeking Temperatures of Maximum Density. *J. Comput. Chem.* **1998,** *19*.
21. Hess, B.; Bekker, H.; Berendsen, H.; Fraaije, J., LINCS: A Linear Constraint Solver for Molecular Simulations. *J. Comput. Chem.* **1998,** *18*.
22. Darden, T. A.; York, D. M.; Pedersen, L. G., Particle Mesh Ewald: An N·log(N) Method for Ewald Sums in Large Systems. *J. Chem. Phys.* **1993,** *98*, 10089-10092.
